# How the Immune System Recognizes the Myriad of Antigens: Diversity of MHC (HLA) and TCR Molecules

**DOI:** 10.14789/ejmj.JMJ25-0044-R

**Published:** 2026-02-06

**Authors:** HISAYA AKIBA

**Affiliations:** 1 Department of Immunology, Faculty of Medicine and Graduate School of Medicine, Juntendo University, Tokyo, Japan; 1 Department of Immunology, Faculty of Medicine and Graduate School of Medicine, Juntendo University, Tokyo, Japan

**Keywords:** MHC, HLA, TCR, antigen-presenting cell, T cell

## Abstract

The immune system must distinguish a vast diversity of pathogens while avoiding harmful responses to self. This specificity is achieved through two unique families of molecules: T-cell receptors (TCRs) on T lymphocytes and major histocompatibility complex (MHC) proteins, known as the human leukocyte antigen molecules in humans, on antigen-presenting cells. MHC molecules provide the structural platform for displaying short peptide fragments derived from intracellular or extracellular proteins. MHC class I molecules present endogenous peptides to CD8^+^ cytotoxic T cells, while MHC class II molecules present exogenous peptides to CD4^+^ helper T cells. The extraordinary diversity of MHC genes, generated through polymorphism and codominant expression, enables a single individual to present an enormous range of peptides, thereby enhancing immune surveillance. However, it also creates barriers to organ transplantation. TCRs, by contrast, achieve their diversity through somatic gene rearrangements. Random recombination of variable (V), diversity (D), and joining (J) gene segments, along with junctional modifications mediated by RAG proteins and terminal deoxynucleotidyl transferase, produces a nearly limitless TCR repertoire. During thymic development, positive selection ensures recognition of self-MHC, while negative selection eliminates autoreactive clones, establishing central tolerance. Together, the polymorphism of MHC molecules and the genetic recombination of TCR genes equip the immune system with the capacity to recognize countless foreign antigens while maintaining self-tolerance. This dual system exemplifies the elegant molecular coevolution of antigen presentation and recognition, forming the foundation of adaptive immunity.

## Introduction

The immune system faces the formidable task of distinguishing a nearly limitless variety of foreign molecules, ranging from viruses and bacteria to parasites and tumor-derived antigens. Unlike innate immunity, which relies on germline-encoded receptors with limited specificity, adaptive immunity has evolved mechanisms to generate a vast repertoire of antigen receptors. This capacity allows the immune system not only to recognize diverse pathogens but also to establish long-lasting memory responses.

Two unique classes of proteins orchestrate antigen recognition in T cells: the major histocompatibility complex (MHC) proteins, known as the human leukocyte antigen (HLA) system in humans, and T-cell receptors (TCRs). MHC molecules are responsible for displaying peptide fragments derived from pathogens or self-proteins on the surface of antigen-presenting cells. TCRs, expressed on T lymphocytes, recognize these peptide-MHC complexes and initiate downstream immune signaling.

The interaction between MHC molecules and TCRs is central to adaptive immunity. The diversity of MHC molecules and TCRs, generated through polymorphism and somatic gene rearrangements, respectively, provides the structural and functional determinants for antigen recognition. Importantly, the immune system must balance this diversity with tolerance, eliminating or suppressing autoreactive T cells. This review focuses on these two molecular systems, highlighting how their structures and mechanisms enable the immune system to cope with the myriads of antigens encountered throughout life.

## The structure and function of MHC （HLA） molecules

**Figure 1:**
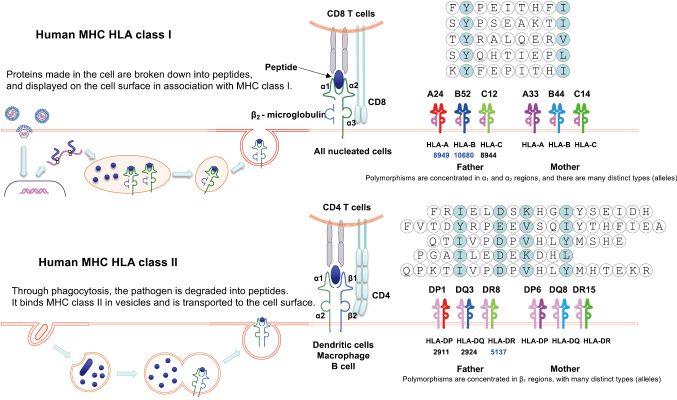
Graphic summary of the structure, function and diversity of MHC (HLA) molecules

The first essential component of antigen recognition is the MHC^1-4)^. In humans, these molecules are encoded within the HLA locus on chromosome 6. MHC molecules are glycoproteins expressed on the cell surface that bind peptide fragments and present them to TCRs. Their structural features, expression patterns, and genetic diversity are crucial for adaptive immunity (Figure 1).

MHC molecules are divided into two primary classes: class I and class II. MHC class I molecules are expressed on nearly all nucleated cells. They consist of a heavy α-chain (with α1, α2, and α3 domains) associated with β2-microglobulin. The peptide-binding groove is formed by the α1 and α2 domains, which typically accommodate peptides 8-9 amino acids long. Class I molecules present endogenous peptides, such as those derived from viral or tumor proteins, to CD8^+^ cytotoxic T cells.

MHC class II molecules are expressed mainly on professional antigen-presenting cells (APCs), including dendritic cells, macrophages, and B cells. They consist of an α-chain (α1 and α2) and a β- chain (β1 and β2). The peptide-binding groove is open at both ends, allowing peptides of 12-20 amino acids. Class II molecules present exogenous peptides to CD4^+^ helper T cells, which then orchestrate broader immune responses.

A defining feature of MHC molecules is their genetic polymorphism. Unlike TCRs, which derive diversity from somatic recombination, MHC diversity is encoded in the germline. Multiple isotypes (HLA-A, HLA-B, HLA-C for class I; HLA-DR, HLA-DQ, HLA-DP for class II) exist, and within each isotype, there are numerous allelic variants. Importantly, MHC alleles are expressed codominantly, so individuals inherit and express alleles from both parents.

This extensive polymorphism ensures that within a population, the ability to present a wide array of peptides is maximized, protecting the species from pathogen escape. However, it also introduces clinical challenges such as graft rejection in organ transplantation, where mismatched HLA molecules are recognized as foreign.

## Mechanisms of antigen presentation

The immune system employs distinct pathways to generate peptide-MHC complexes, reflecting the origin of the antigens^5-8)^.

MHC class I pathway (endogenous antigens): proteins synthesized within the cytosol, including viral proteins, are degraded by the proteasome into short peptide fragments. These peptides are transported into the endoplasmic reticulum (ER) by the transporter associated with antigen processing (TAP). In the ER, peptides are loaded onto newly synthesized MHC class I molecules, assisted by chaperone proteins. The peptide-MHC class I complexes are then trafficked through the Golgi apparatus to the cell surface. This process ensures that any cell harboring intracellular pathogens can be identified and destroyed by CD8^+^ cytotoxic T cells.

MHC class II pathway (exogenous antigens): professional APCs internalize extracellular pathogens via endocytosis or phagocytosis. The ingested proteins are degraded in endosomal and lysosomal compartments into peptides. MHC class II molecules, synthesized in the ER, are initially bound by an invariant chain (Ii) that prevents premature peptide binding. In the endosomal pathway, the Ii is degraded, leaving a small fragment (CLIP) that is subsequently exchanged for antigenic peptides with the help of HLA-DM. The resulting peptide- MHC class II complexes are transported to the cell surface for recognition by CD4^+^ T cells.

A specialized mechanism known as cross-presentation allows specific dendritic cells to present extracellular antigens via MHC class I molecules. This cross-presentation is critical for initiating CD8^+^ T-cell responses against viruses that do not directly infect APCs.

Together, these antigen presentation pathways ensure that T cells survey both intracellular and extracellular compartments, maintaining comprehensive immune surveillance.

## Polymorphism and diversity of MHC (HLA)

Unlike TCRs, whose diversity is generated by somatic recombination, the diversity of MHC molecules is encoded in the germline. This diversity arises through two main mechanisms: polymorphism and multiplicity^9-10)^.

Polymorphism refers to the existence of numerous allelic variants within the population. For example, over 10,680 alleles have been identified for HLA-B, each differing in amino acid residues within the peptide-binding groove^11)^. These polymorphisms alter peptide specificity by dictating which residues serve as anchoring positions. As a result, different individuals can present different sets of peptides, even from the same pathogen.

Multiplicity refers to the fact that multiple MHC isotypes are expressed simultaneously. Humans have three classical class I genes (HLA-A, HLA-B, and HLA-C) and three classical class II genes (HLA-DR, HLA-DQ, and HLA-DP). Because expression is codominant, each individual expresses both maternal and paternal alleles, yielding up to six class I molecules and a comparable number of class II molecules. This codominance dramatically increases the peptide-presenting capacity of each individual.

The evolutionary advantage of this diversity lies in its contribution to population-level survival. While an individual may be susceptible to pathogens that evade recognition by their specific HLA alleles, the species retains resistance because of allelic diversity. However, this diversity creates clinical complications. In organ transplantation, mismatched HLA molecules are potent targets of T-cell responses, leading to graft rejection unless immunosuppressive strategies are employed. Furthermore, specific HLA alleles are associated with autoimmune diseases, including HLA-B27 in ankylosing spondylitis and HLA-DR4 in rheumatoid arthritis, highlighting the delicate balance between pathogen defense and autoimmunity.

## The structure and function of T-Cell receptors

**Figure 2:**
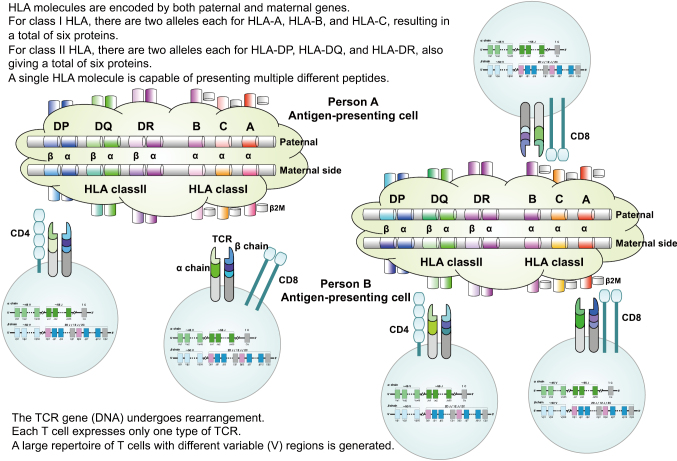
Graphic summary of the structure, function and diversity of TCR

T-cell receptors (TCRs) are cell surface glycoproteins that mediate the recognition of peptide- MHC complexes^12-14)^. Unlike antibodies, which can be secreted and recognize native conformational epitopes, TCRs are membrane-bound and only recognize peptides presented by MHC molecules (Figure 2).

Most T cells express αβ TCRs, composed of two chains (TCRα and TCRβ) linked by disulfide bonds. Each chain has a variable (V) domain responsible for antigen recognition and a constant (C) domain. The antigen-binding site is formed by the variable regions of both α and β chains, particularly the complementarity-determining regions (CDRs). These hypervariable loops directly interact with the peptide presented by MHC. A minority of T cells express γδ TCRs, which recognize non-peptide antigens such as lipids and phosphorylated molecules; however, most adaptive immune responses are mediated by αβ T cells.

The TCR itself lacks signaling motifs in its short cytoplasmic tails. Antigen recognition signals are therefore transduced through the associated CD3 complex, composed of γε, δε, and ζζ dimers. These CD3 subunits contain immunoreceptor tyrosine- based activation motifs (ITAMs) that initiate signaling cascades upon antigen binding. Collectively, a TCR-CD3 complex contains ten ITAMs, which amplify antigen recognition, resulting in robust intracellular signaling.

Specificity in antigen recognition is defined by MHC restriction. CD4^+^ T cells recognize peptides bound to MHC class II molecules, whereas CD8^+^ T cells recognize peptides bound to MHC class I molecules. This dual recognition ensures that helper T cells are primarily responding to extracellular pathogens, whereas cytotoxic T cells focus on intracellular infections.

## Generation of TCR diversity

The enormous diversity of the TCR repertoire is a defining feature of adaptive immunity^15-18)^. This diversity is not inherited in the germline but generated de novo in developing T cells within the thymus through a process known as V(D)J recombination.

The TCR α-chain locus contains numerous variable (V) and joining (J) segments, while the β- chain locus contains V, diversity (D), and J segments. During thymocyte development, random recombination events bring together a single V, (D), and J segment, which are then joined to a constant (C) region. This DNA rearrangement is catalyzed by the recombination-activating gene (RAG) products RAG1 and RAG2, which introduce double-stranded breaks at recombination signal sequences (RSSs) flanking each gene segment.

In addition to the combinatorial diversity, junctional diversity further expands the repertoire. At the recombination junctions, nucleotides may be deleted or added randomly, usually mediated by terminal deoxynucleotidyl transferase (TdT). These modifications create unique sequences in the CDR3, the most variable and antigen-contacting region of the TCR.

Collectively, these processes can theoretically generate an enormous number of distinct TCRs, although the actual number of circulating T cells is much smaller. Importantly, each T cell expresses only one TCR specificity owing to allelic exclusion, ensuring clonal specificity in immune responses.

This immense diversity enables the immune system to recognize virtually any peptide-MHC complex. However, it poses the challenge of self- reactivity, which must be controlled by subsequent thymic selection mechanisms.

## Thymic selection and central tolerance

The process of generating TCR diversity through random gene rearrangements inevitably produces many receptors that are either nonfunctional or autoreactive. To shape a functional and self-tolerant T-cell repertoire, developing thymocytes undergo rigorous selection in the thymus, an organ located in the anterior mediastinum above the heart^19-23)^.

Thymic development proceeds through distinct stages. Early progenitors arising from the bone marrow migrate to the thymus as double-negative (DN) cells, which lack CD4 and CD8 expression. Upon successful rearrangement of the TCR genes, thymocytes progress to the double-positive (DP) stage, expressing both CD4 and CD8 molecules along with their newly formed TCRs. At this point, thymocytes face two critical checkpoints: positive selection and negative selection.

Positive selection occurs in the thymic cortex and is primarily mediated by cortical epithelial cells that express self-MHC molecules. DP thymocytes that can recognize self-MHC molecules with at least a minimal affinity receive survival signals, while those that fail to interact undergo apoptosis. This process establishes MHC restriction, ensuring that mature T cells will only respond to antigens presented by the host's own MHC molecules.

Negative selection occurs mainly in the medulla and eliminates autoreactive thymocytes. Dendritic cells and medullary epithelial cells present a vast array of self-peptides, including tissue-specific antigens expressed under the control of the autoimmune regulator (AIRE) transcription factor. Thymocytes with high-affinity recognition of self- peptide-MHC complexes are induced to undergo apoptosis, preventing the emergence of strongly autoreactive clones.

The outcome of thymic selection is the generation of single-positive (SP) thymocytes that express either CD4 or CD8, corresponding to recognition of MHC class II or class I molecules, respectively. These cells exit the thymus as mature naïve T cells capable of responding to foreign antigens but tolerant of self. Failures in these processes can lead to autoimmunity, immunodeficiency, or lymphoproliferative disease.

## T cell activation, differentiation, and peripheral tolerance

Naïve T cells exiting the thymus enter secondary lymphoid organs, recirculating through lymph nodes and spleen. Antigen presentation is mediated by dendritic cells (DCs), which capture pathogens in tissues, migrate to lymphoid sites, and present processed peptides bound to MHC molecules. Mature DCs provide both antigen and costimulatory signals, making them the most potent activators of naïve T cells^24-26)^.

Activation requires two distinct signals. First, the TCR binds a peptide-MHC complex. Second, costimulatory interactions occur, primarily CD28 on T cells engaging B7 molecules (CD80/86) on APCs. Without costimulation, T cells undergo anergy, a state of functional unresponsiveness that contributes to the development of tolerance. Following activation, the secretion of IL-2 and the expression of high-affinity IL-2 receptors drive clonal expansion. CTLA-4, expressed on activated T cells, competes with CD28 for B7 binding, limiting proliferation and providing an inhibitory checkpoint.

CD8^+^ T cells differentiate into cytotoxic T lymphocytes (CTLs), which eliminate virus-infected or tumor cells through perforin- and granzyme-mediated apoptosis, or via the FasL and TNF pathways. Effector CTLs act without costimulatory input, ensuring rapid clearance of infected cells.

CD4^+^ T cells adopt specialized fates according to cytokine cues. Exposure to IL-12 promotes differentiation into Th1 cells, which secrete IFN-γ and TNF-α to activate macrophages. In the presence of IL-4, Th2 cells arise and secrete IL-4, IL-5, and IL-13, thereby activating B cells and antibody production. When TGF-β, IL-6, and IL-23 interact, naïve CD4^+^ cells develop into Th17 cells that secrete IL-17 and IL-22, thereby mediating the recruitment of neutrophils, particularly at mucosal barriers. T follicular helper cells (Tfh) emerge under the influence of IL-6 and IL-21 and orchestrate germinal center formation and antibody affinity maturation. By contrast, regulatory T cells (Tregs), induced by TGF-β and IL-2, limit autoreactive T cells through the production of inhibitory cytokines, including IL-10 and TGF-β.

A key element of immune balance is peripheral tolerance, which complements central tolerance established in the thymus. While negative selection removes many autoreactive clones, some escape into the periphery^27)^. Peripheral tolerance mechanisms prevent these cells from causing autoimmunity. The first mechanism is anergy, wherein recognition of self-antigen without costimulation renders T cells inert. The second involves suppression by Treg cells, which inhibit effector T cells and dampen inflammation, maintaining homeostasis. The third is activation-induced cell death (AICD), which eliminates overly persistent or autoreactive clones through the Fas-FasL signaling pathway.

The importance of peripheral tolerance is underscored by its failure: defects in Treg development, CTLA-4 function, or AICD are associated with autoimmune diseases, including type 1 diabetes, lupus, and multiple sclerosis. Thus, while effector CD8 and CD4 T cells protect against infection and cancer, equally critical is the restraint imposed by peripheral tolerance, which ensures that immune defense does not become self-destructive.

In summary, T-cell immunity integrates antigen recognition, costimulation, cytokine-driven differentiation, and tolerance checkpoints. Effector subsets provide specialized protection, while peripheral tolerance mechanisms, including anergy, Treg suppression, and cell death, are indispensable for preventing autoimmune pathology. Together, these systems maintain the delicate equilibrium between effective host defense and self-tolerance.

## Conclusion

The capacity of the immune system to recognize a myriad of antigens rests on two fundamental pillars: the polymorphism of MHC molecules and the somatic recombination of TCR genes. MHC molecules provide structural a framework for antigen display, whereas TCRs supply the specificity for antigen recognition. Thymic selection shapes a repertoire that is both self-MHC restricted and self-tolerant.

This dual system ensures comprehensive immune surveillance, striking a balance between responsiveness to pathogens and tolerance to self. Beyond basic biology, the implications of MHC and TCR diversity extend to transplantation, autoimmunity, infectious disease, and cancer therapy. The study of these molecules reveals not only the molecular logic of adaptive immunity but also offers avenues for therapeutic innovation. As immunology advances, the integration of genomics, structural biology, and clinical research will deepen our understanding of how MHC and TCR diversity influence health and disease.

## Author contributions

HA wrote and checked the manuscript.

## Conflicts of interest statement

The author declare that there are no conflicts of interest.
